# Identification of Novel Sensitive and Reliable Serovar-Specific Targets for PCR Detection of *Salmonella* Serovars Hadar and Albany by Pan-Genome Analysis

**DOI:** 10.3389/fmicb.2021.605984

**Published:** 2021-03-16

**Authors:** Qinghua Ye, Yuting Shang, Moutong Chen, Rui Pang, Fan Li, Xinran Xiang, Chufang Wang, Baoqing Zhou, Shuhong Zhang, Jumei Zhang, Xiaojuan Yang, Liang Xue, Yu Ding, Qingping Wu

**Affiliations:** ^1^Guangdong Provincial Key Laboratory of Microbial Safety and Health, State Key Laboratory of Applied Microbiology Southern China, Institute of Microbiology, Guangdong Academy of Sciences, Guangzhou, China; ^2^Department of Food Science & Technology, Jinan University, Institute of Food Safety & Nutrition, Jinan University, Guangzhou, China

**Keywords:** *Salmonella*, C2 serogroups, serovar-specific molecular targets, PCR, pan-genome analysis

## Abstract

The accurate and rapid classification of *Salmonella* serovars is an essential focus for the identification of isolates involved in disease in humans and animals. The purpose of current research was to identify novel sensitive and reliable serovar-specific targets and to develop PCR method for *Salmonella* C2 serogroups (O:8 epitopes) in food samples to facilitate timely treatment. A total of 575 genomic sequences of 16 target serovars belonging to serogroup C2 and 150 genomic sequences of non-target serovars were analysed by pan-genome analysis. As a result, four and three specific genes were found for serovars Albany and Hadar, respectively. Primer sets for PCR targeting these serovar-specific genes were designed and evaluated based on their specificity; the results showed high specificity (100%). The sensitivity of the specific PCR was 2.8 × 10^1^–10^3^ CFU/mL and 2.3 × 10^3^–10^4^ CFU/mL for serovars Albany and Hadar, respectively, and the detection limits were 1.04 × 10^3^–10^4^ CFU/g and 1.16 × 10^4^–10^5^ CFU/g in artificially contaminated raw pork samples. Furthermore, the potential functions of these serovar-specific genes were analysed; all of the genes were functionally unknown, except for one specific serovar Albany gene known to be a encoded secreted protein and one specific gene for serovars Hadar and Albany that is a encoded membrane protein. Thus, these findings demonstrate that pan-genome analysis is a precious method for mining new high-quality serovar-targets for PCR assays or other molecular methods that are highly sensitive and can be used for rapid detection of *Salmonella* serovars.

## Introduction

*Salmonella* is one of crucial foodborne pathogen that causes illness worldwide, including diarrhoea, gastroenteritis, typhoid, paratyphoid, septicaemia, and other clinical syndromes (Dekker and Frank, 2015). Pigs, poultry and their eggs, and cattle could be infected by *Salmonella* (Rodriguez et al., 2006; Xu et al., 2017), which can be disseminate to humans via ingestion of contaminated pork, chicken, eggs, beef, and milk (Kerouanton et al., 2013; Bonardi, 2017). Salmonellosis represents a serious occupational and public health hazard. Characteristic lipopolysaccharide, which is composed of lipid-A and a major O-antigen with side chains of repeating units of sugar residues, is a dominant cause for Salmonellosis (Sannigrahi et al., 2020). On the basis of the O antigen, *Salmonella* has been divided into 46 serogroups that markedly differ in their virulence (Davies et al., 2013); the isolates of D, B, C1, C2, and E serogroups take up a great majority of foodborne outbreaks (Graziani et al., 2017). Several studies have undertaken mechanism analysis and developed detection methods for *Salmonella* serovars Enteritidis (Wagner and Hensel, 2011), Typhimurium (Maurischat et al., 2015), and Derby (Castelijn et al., 2013), since they top the list of the most prevalent serotypes (Ni et al., 2018). However, little information about the other serovars is available, particularly for the infective serotypes that belong to the C2 serogroup. For example, S. Hadar is a host-non-specific serotype that causes infection in both humans and animals. It has been identified previously in hospital outbreaks and confirmed as the fourth most frequently isolated *Salmonella* serovar in Germany (Weidebotjes et al., 1998; Deshpande et al., 2015). Furthermore, instances of multidrug resistance in S. Albany have increased in recent years (Doublet et al., 2003). In order to reduce the prevalence of *Salmonella*, establishment rapid and feasible detection methods is essential for the identification of high-risk *Salmonella* serovars.

Traditional serotyping methods are based on slide and tube agglutination tests using O and H antigen-specific anti-sera (Herrera-León et al., 2007). However, these measures are costly, labour-intensive, time-consuming and insensitive, with certain isolates remaining partially typed or untyped, attributed to the loss of somatic and flagellar antigens. Nucleic acid amplification tests (NAATs), with the advantages of their high-speed, convenient operation, high-sensitivity and-specificity, have been extensively applied in different fields including the food industry, agriculture, and environmental sciences (Lee et al., 2019). Selection of appropriate pathogen genes is key to the sensitivity and specific detection capabilities of NAATs. Various genes have been used to detect *Salmonella* serogroups and serovars including *SNSL254_A2005* for *Salmonella* C2 serogroups (Liu et al., 2011), *STM4495* for S. Typhimurium (Liu et al., 2012), *sdfI* for S. Enteritidis (De Freitas et al., 2010), *ISR2* for S. Infantis (Akiba et al., 2011), and *Newp2* for S. Newport (Bugarel et al., 2017)—some of which may also be present in non-target strains.

With the recent advancements in high-throughput sequencing technology, an increasing number of whole genome sequences for *Salmonella* are available online. Through bioinformatics analysis, the resulting genomic information elucidates the diversity of *Salmonella* serovars. The pan-genome is the sum of a core genome which constitutes of genes present in all of the strains analysed and a dispensable genome that comprises genes present in some but not all of the strains analysed (Tettelin et al., 2008; Kim et al., 2020). We obtain molecular targets by selecting the co-existence genes in the target serovar of *Salmonella* and excluding the unspecific genes that are distributed among non-target serovars. Based on our prior research on the prevalence of *Salmonella* in China (Yang et al., 2015a, b, 2016, 2020a, b), more than 1,400 strains of *Salmonella* with 42 serovars have been isolated. We undertook the present study with the goal of identifying serovar-specific molecular targets for the most common serovars in serogroup C2 by pan-genome analysis of *Salmonella* genome sequences. Furthermore, practical PCR verification was conducted with a large number of bacterial strains and the specificity, sensitivity, and reliability of the PCR were also assessed. We evaluated the specificity targets of various *Salmonella* serovars by PCR and analysed the potential functions of these target genes. These data were analysed to obtain high-quality candidates for NAATs, as the use of this PCR-typing method permits for the identification of *Salmonella* serovars can be save a large abundance of time and money, and its accuracy is the same as the traditional serotyping methods.

## Materials and Methods

### Tested Strains

One hundred fifty-eight *Salmonella* strains which were serotyped by traditional methods, and twenty-two non-Salmonella strains were used for assessing the specificity in our study (Table 1). Sixteen and four strains were acquired from The American Type Culture Collection (ATCC, Manassas, VA, United States) and National Centre for Medical Culture Collection (CMCC, Beijing, China), and other strains were isolated from food sources in our laboratory from 2011 to 2014.

**TABLE 1:** Bacteria strains and specificity of PCR in this study.

			PCR results using 7 novel serovar-specific targets^&^						
Species/Serotype	Strain*	Number of strains tested	1	2	3	4	5	6	7
S. Albany	Laboratory strain	17	+	+	+	+	–	–	–
S. Hadar	Laboratory strain	5	–	–	–	–	+	+	+
S. Typhimurium	ATCC14028	1	–	–	–	–	–	–	–
S. Typhimurium	Laboratory strain	9	–	–	–	–	–	–	–
S. Derby	Laboratory strain	9	–	–	–	–	–	–	–
S. Indiana	Laboratory strain	9	–	–	–	–	–	–	–
S. Agona	Laboratory strain	5	–	–	–	–	–	–	–
S. Enteritidis	CMCC50335	1	–	–	–	–	–	–	–
S. Enteritidis	Laboratory strain	9	–	–	–	–	–	–	–
S. Weltevreden	Laboratory strain	9	–	–	–	–	–	–	–
S. London	Laboratory strain	8	–	–	–	–	–	–	–
S. Wandsworth	Laboratory strain	1	–	–	–	–	–	–	–
S. Stanley	Laboratory strain	1	–	–	–	–	–	–	–
S. Rissen	Laboratory strain	9	–	–	–	–	–	–	–
S. Meleagridis	Laboratory strain	8	–	–	–	–	–	–	–
S. Corvallis	Laboratory strain	1	–	–	–	–	–	–	–
S. Kottbus	Laboratory strain	1	–	–	–	–	–	–	–
S. Pomona	Laboratory strain	9	–	–	–	–	–	–	–
S. Senftenberg	Laboratory strain	9	–	–	–	–	–	–	–
S. Braenderup	Laboratory strain	9	–	–	–	–	–	–	–
S. Tallahassee	Laboratory strain	1	–	–	–	–	–	–	–
S. Newport	Laboratory strain	1	–	–	–	–	–	–	–
S. Potsdam	Laboratory strain	1	–	–	–	–	–	–	–
S. Infantis	Laboratory strain	5	–	–	–	–	–	–	–
S. Muenster	Laboratory strain	1	–	–	–	–	–	–	–
S. Manhattan	Laboratory strain	1	–	–	–	–	–	–	–
S. Kentucky	Laboratory strain	1	–	–	–	–	–	–	–
S. Chailey	Laboratory strain	1	–	–	–	–	–	–	–
S. Litchfield	Laboratory strain	1	–	–	–	–	–	–	–
S. Bareilly	Laboratory strain	1	–	–	–	–	–	–	–
S. Give	Laboratory strain	1	–	–	–	–	–	–	–
S. Montevideo	Laboratory strain	1	–	–	–	–	–	–	–
S. Mbandaka	Laboratory strain	1	–	–	–	–	–	–	–
S. Riggil	Laboratory strain	1	–	–	–	–	–	–	–
S. Lomita	Laboratory strain	1	–	–	–	–	–	–	–
S. Saintpaul	Laboratory strain	1	–	–	–	–	–	–	–
S. Aberdeen	Laboratory strain	1	–	–	–	–	–	–	–
S. Istanbul	Laboratory strain	1	–	–	–	–	–	–	–
S. Lagos	Laboratory strain	1	–	–	–	–	–	–	–
S. Singapore	Laboratory strain	1	–	–	–	–	–	–	–
S. Eingedi	Laboratory strain	1	–	–	–	–	–	–	–
S. Virchow	Laboratory strain	3	–	–	–	–	–	–	–
S. Heidelberg	Laboratory strain	1	–	–	–	–	–	–	–
S. Thompson	Laboratory strain	1	–	–	–	–	–	–	–
*E. coli*	ATCC25922	1	–	–	–	–	–	–	–
*E. coli*	CMCC44105	1	–	–	–	–	–	–	–
*E. coli O157*	ATCC 12900	1	–	–	–	–	–	–	–
*E. coli*	ATCC43886	1	–	–	–	–	–	–	–
*C. sakazakii*	ATCC 29544	1	–	–	–	–	–	–	–
*C. sakazakii*	3414c1	1	–	–	–	–	–	–	–
*S. sonnei*	CMCC(B)51592	1	–	–	–	–	–	–	–
*Y. enterocolitica*	Laboratory strain	1	–	–	–	–	–	–	–
*Y. enterocolitica*	CMCC 52204	1	–	–	–	–	–	–	–
*V. parahemolyticus*	ATCC 33847	1	–	–	–	–	–	–	–
*V. parahemolyticus*	ATCC 17802	1	–	–	–	–	–	–	–
*S. aureus*	ATCC25923	1	–	–	–	–	–	–	–
*S. aureus*	ATCC29213	1	–	–	–	–	–	–	–
*P. aeruginosa*	ATCC 9027	1	–	–	–	–	–	–	–
*P. aeruginosa*	ATCC 15442	1	–	–	–	–	–	–	–
*B. cereus*	ATCC14579	1	–	–	–	–	–	–	–
*L. monocytogenes*	ATCC19115	1	–	–	–	–	–	–	–
*L. monocytogenes*	CMCC 54104	1	–	–	–	–	–	–	–
*B. subtilis*	ATCC6633	1	–	–	–	–	–	–	–
*B. mycoides*	ATCC10206	1	–	–	–	–	–	–	–
*C. jejuni*	Laboratory strain	2	–	–	–	–	–	–	–
Total	180								

*Laboratory strain that were isolated from food samples. ^&^1–4: group_20134, group_22774, group_29844 and group_29846 (for serovar Hadar-specific gene); 5–7: group_27286, group_27289 and group_27297 (for serovar Albany-specific gene).

### Genomic Sequences of *Salmonella* Species

Considering the number of scaffolds exceed 200, the genome sequence presumably has large gaps. Thus, the number of scaffold less than or equal to 200 for representative strains of the genome sequences are applied to analysis in this study. The 16 serovars belonging to serogroup C2 were represented by 575 isolates, with 1–248 isolates for each serovar. An additional 150 isolates from non-target serovars were also included. All 725 *Salmonella* genomes were downloaded from NCBI GenBank (NIH, Bethesda, MD, United States) in nucleotide FASTA format. A complete listing of the 725 genomes including GenBank identifier, serogroup, serovar, isolation source, geographic location, collection date, genome size, GC%, the number of scaffold etc. are listed in Supplementary Table 1.

### Phylogenetic Analysis

The core-genome alignment of *Salmonella* was implemented using Harvest v1.1.2 software (Treangen et al., 2014) with the S. Albany ATCC 51960 genome as a reference. Conducting recombination and removing the putative recombined regions were implemented Genealogies Unbiased By recomBinations In Nucleotide Sequences (Gubbins, Croucher et al., 2015). Single nucleotide polymorphisms (SNPs) data were obtained from the recombination-free core-genome alignment by the script online at https://github.com/sanger-pathogens/snp-sites. The maximum-likelihood (ML) phylogenetic tree was established on the connected core SNPs by Random Axelerated Maximum Likelikhood (RAxML v8.2.10) in the GTRGAMMA model (1000 bootstrap) (Stamatakis, 2014). Results were visualised with Interaction Tree Of Life (iTOL, Letunic and Bork, 2016).

### Pan-Genome Analysis and Identification of Serovar-Specific Genes

All analysed genome sequences were re-annotated using Prokka v1.11 (Seemann, 2014). With a BlastP (NCBI/NIH) identity cut-off of 70%, the Prokka output was utilised to establish the pan-genome by Roary v3.11.2 (Page et al., 2015). The absence/presence profile of all genes across all samples was transferred into a 0/1 matrix with a local script. Based on the 0/1 matrix, the core genes (presenting in over 99% *Salmonella* genomes) and serovar-specific genes were screened, presenting in over 95% target serovar strains (considered soft-core genes) and in less than 5% of other serovar strains (considered non-related) (Buchanan et al., 2017; Thépault et al., 2017). Functional classification of the core genes were assigned to the Gene Ontology (Go) terms (Ashburner et al., 2000). The specificity of serovar-specific genes were further confirmed by BLAST against the NCBI nucleotide sequence database.

### Functional Analysis of Serovar-Specific Genes

The serovar-specific genes were annotated in public databases including the Non-redundant (NR), Kyoto Encyclopaedia of Genes and Genomes (KEGG), Clusters of Orthologous Groups (COG), Virulence Factors Database (VFDB), Comprehensive Antibiotic Resistance Database (CARD), ResFinder, and Antibiotic Resistance Gene-ANNOTation (AGR-ANNOT). TMHMM Server, SignalP, and Phobius were also implemented to confirm whether these genes encode membrane and/or secretory proteins.

### Evaluation of Specificity and Sensitivity for Novel Serovar-Specific Molecular Targets

The novel serovar-specific target genes were applied to design primer sets (Table 2) using Oligo 7 software and synthesised by Generay Biotech Co., Ltd. (Shanghai, China). The specificity of the primer sets were tested against the 180 strains listed in Table 1. Genomic DNA was extracted using the DNeasy blood and tissue kit (Qiagen, Shanghai, China) according to the manufacturer’s instructions. The purity and concentration of 50 μL genomic DNA samples were measured by Qubit^®^ 3.0 Fluorometer (Life Invitrogen, United States) and stored at -20°C before being used as a template for PCR. Each 25 μL PCR amplification mixture consisted 12.5 μL of buffer (2×, Novoprotein Scientific Inc., Shanghai, China), 1 μL each of the forward and reverse primer (5 μM), 1 μL of template DNA, and sterile distilled water (filled to a final volume). The PCR conditions were 95°C for 5 min, followed by 35 cycles of 95°C for 30 s, 60°C for 30 s, and 72°C for 30–60 s, and final extension at 72°C for 10 min. The PCR products were analyse via 1.5% agarose gel electrophoresis and visualised using a UV transilluminator (GE 138 Healthcare, WI, United States).

**TABLE 2:** Specific primer sets and sensitivity of PCR for artificially contaminated raw pork samples with *Salmonella* serovar Hadar and Albany.

Serotype	Gene location^X^	Gene	Sequences (5′-3′)	PCR product (bp)	Detection limit for artificially pork sample (CFU/g)
*S. Hadar*	3719826–3721526	group_20134	TTGATCTGCTGCTGCCTAAT	1,604	1.16 × 10^5^
			TGGAACTGGTGTCCTGAAAT		
	3715592–3716611	group_22774	GGAATAACAAAGGTGGTACT	902	1.16 × 10^4^
			CCTGACCTTAGAGAATGGCT		
	3717735–3717890	group_29844	TGCCTGTGAGTTTTAACTCT	155	116 × 10^5^
			CTATGTCTCAGCCAGTTCAT		
	3721669–3722367	group_29846	GCGTACCACATCAAATCAGT	567	1.16 × 10^4^
			CCCAGAGACATGCCAAAAAT		
*S. Albany*	3472553–3473584	group_27286	CTCAGTTACCAGAAAGAAGT	922	1.04 × 10^3^
			GAAGCCTGTTATTGATGAGT		
	3608857–3610113	group_27289	GCGTTGAGGTTGAGTGGTTG	1,187	1.04 × 10^4^
			GAACAGCAAATCACGGTAGT		
	4265383–4265706	group_27297	TCCTACAAGCTTTGGCGAAT	283	1.04 × 10^3^
			GTGGCAACGGAACTTAAGAC		

*^X^Reference strain are S. Hadar str. FDAARGOS_313 and S. Albany str. ATCC 51960.*

For sensitivity testing, 10-fold serial dilutions (10^7^–10^0^ CFU/mL) of S. Hadar strain SA39(5) and S. Albany strain SA48(32) were subjected to DNA extraction. The PCR amplification were implemented as mentioned above except 2 μL of each genome DNA was used as a template.

### Artificial Contamination of Raw Pork Samples

The S. Hadar strain SA39(5) and S. Albany strain SA48(32) were enriched in LB broth at 37°C overnight, and the 10-fold serial dilutions of different concentrations of cultures were prepared. Twenty-five grams of pork determined to be negative for *Salmonella* by standard culture methods was homogenised in 225 mL of sterile saline to obtain the matrix. Subsequently, 9 mL homogenate was spiked with 1 mL of various concentrations of a specific *Salmonella* serovar to final levels of approximately 10^8^–10^1^ CFU/g samples. DNA was extracted from 1 mL of the mixture and analysed by PCR under the same conditions. Non-inoculated pork meat was used as the negative control and all assays were performed independently, in triplicate.

## Results

### Phylogenetic Analysis of *Salmonella*

**FIGURE 1:**
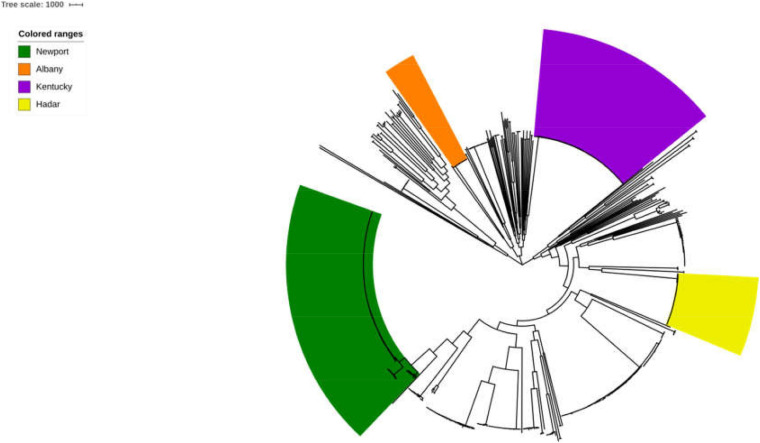
Phylogenetic analysis of *Salmonella*. The ML phylogenetic tree was established based on the connected core 1,803 core-genome SNPs from725 selected *Salmonella* strains. Strains were distributed across serovars and 100% (17/17), 100% (41/41), 80.34% (94/117), and 57.66% (143/248) strains belonged to serovar Albany, Hadar, Kentucky, and Newport are clustered, respectively.

The 725 selected *Salmonella* isolates differed in the 1,803 core-genome SNPs. The ML phylogenetic tree was established based on the connected core SNPs. Isolates were distributed across serovars and all isolates belonged to serovar Albany and Hadar are clustered respectively. Notably, 80.34% (94/117) and 57.66% (143/248) strains belonged to serovar Kentucky and Newport are clustered, respectively (Figure 1). Isolates that fell within different serovars were highly diverse, indicating that an evolutionary difference might exist between these serovars.

### Identification of Serovar-Specific Genes in *Salmonella*

**FIGURE 2:**
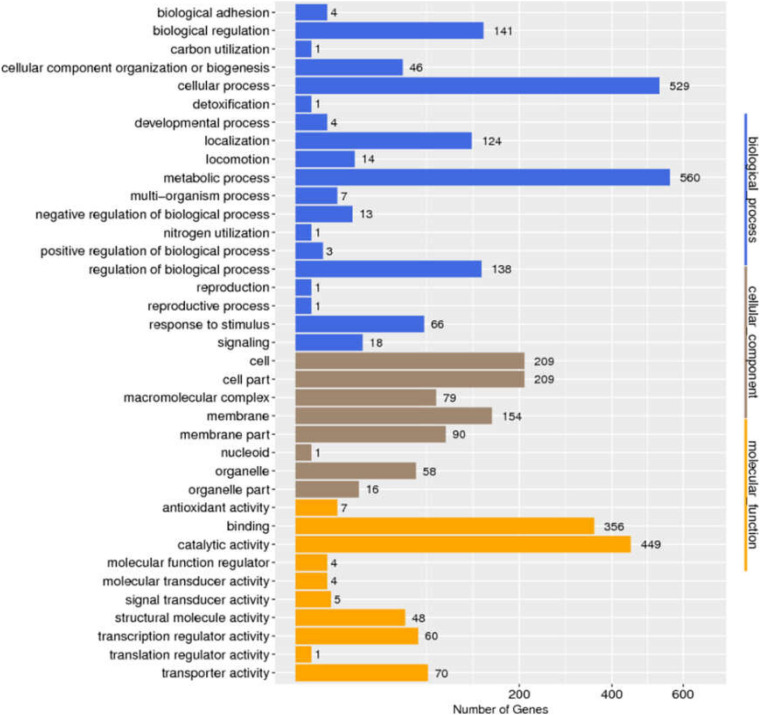
Distribution of GO categories in conserved genes of *Salmonella*.

We determined the size and distribution of the *Salmonella* pan-genome across the 725 genome sequences and the analysis revealed a set of 1,318 conserved genes that were universally present within ≥99% *Salmonella* genomes. Functional profiles of conserved genes were determined in Figure 2. Furthermore, a group of 1,190 genes were present in 95% ≤ strains < 99%, which was called the soft-core genes of *Salmonella*. Besides, 2,904 and 30,032 genes were found in 15% ≤ strains < 95% and 0% ≤ strains < 15% of the isolates of *Salmonella*. Consistent with previous report (Chanda et al., 2020), the *Salmonella* showed an open pan-genome structure, and that the size of pan-genome continue to expand with addition sequenced genomes, but its conserved genes will remain stable (Supplementary Figure S1).

Based on the gene presence/absence profile among the 725 *Salmonella* genomes, 10 fragments were found to be specific to serogroups C2 and serovar Hadar and Albany. For other serovars of Salmonella serogroup C2, there is no novel single targets because the percentage of genomic sequences of strains for the genes presence in target-serovar were less than 85% and in non-target serovars were more than15%. Notably, four (group_20134, group_22774, group_29844, and group_29846) and three (group_27286, group_27289, and group_27297) specific gene markers were found in serovar Hadar and Albany. However, *SNSL254_A2005* is a previously reported marker in serogroups C2 and HSR3 and Hadspe are previously reported markers in Hadar. The percentage of genomic sequences of strains for the present in target and non-target serovars are shown in Table 3.

**TABLE 3:** Presence profile of specific targets for *Salmonella* serogroups C2 and serovars.

Serotype	Related gene	Presence profile		Source
		In target (%)	In non-target (%)	
Serogroup C2	SNSL254_A2005	555 (96.52%)	1 (2%)	Liu et al., 2011
S. Hadar	group_20134	40 (97.56%)	0 (0)	Our study
	group_22774	40 (97.56%)	0 (0)	Our study
	group_29844	40 (97.56%)	0 (0)	Our study
	group_29846	40 (97.56%)	0 (0)	Our study
	HSR3	40 (97.56%)	2 (0.29%)	Chiang et al., 2018
	Hadspe	34 (82.83%)	0 (0)	Bugarel et al., 2017
S. Albany	group_27297	15 (100%)	2 (0.28%)	Our study
	group_27289	15 (100%)	1 (0.14%)	Our study
	group_27286	15 (100%)	1 (0.14%)	Our study

### Characterisation of Serovar-Specific Genes

One serovar albany-specific gene (group_27289) is linked with the function of defence mechanisms (Supplementary Table S2). However, all other serovar-specific genes were annotated uncharacterised functions. We went on to predict whether these genes participate in secretion. According to the prediction pipeline described in previous studies (refs), potentially secreted extracellular proteins and cell membrane proteins were identified from serovar-specific genes (Supplementary Table S2). The group_29846 gene (for serovar Hadar-specific gene) and group_27297 gene (for serovar Albany-specific gene) are encoded membrane proteins. Group _27286 (for serovar albany-specific gene) is encoded as a secreted protein. In addition, the seven specific genes were not related to the virulence-associated genes or the antibiotic resistance-associated genes.

### Specificity and Sensitivity Assessment of Serovar-Specific Genes by PCR

A total of 158 *Salmonella* strains and 22 non-*Salmonella* strains (Table 1) were applied to assess the specificity of the primer sets designed based on serovar-specific fragments by PCR (Table 2). The proper detectable amplicon were observed from *Salmonella* serovar Hadar and Albany strains, but no amplification was obtained with DNA from all non-target strains. Thus, four Hadar-specific and three Albany-specific primer sets were accurately detected of *Salmonella* serovar Hadar and Albany (Table 1).

**FIGURE 3:**
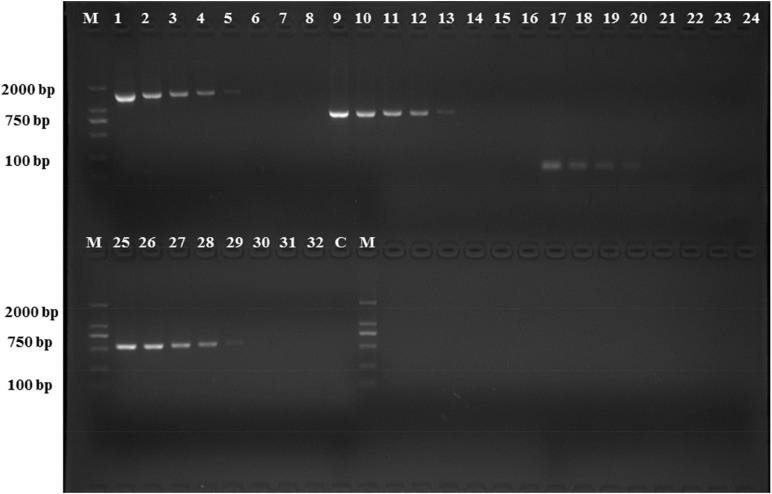
PCR detection sensitivity using dilutions of a pure culture of *S. Hadar* strain SA39(5). Lane M: DSTM 2000 marker; Lane C: negative control (double-distilled H_2_O); lane 1–8, 9–16, 17–24, and 25–32 primer sets based ongroup_20134, group_22774, group_29844, and group_29846 genes, respectively (concentrations ranging from 2.3 × 10^7^ to 10^0^ CFU/mL).

**FIGURE 4:**
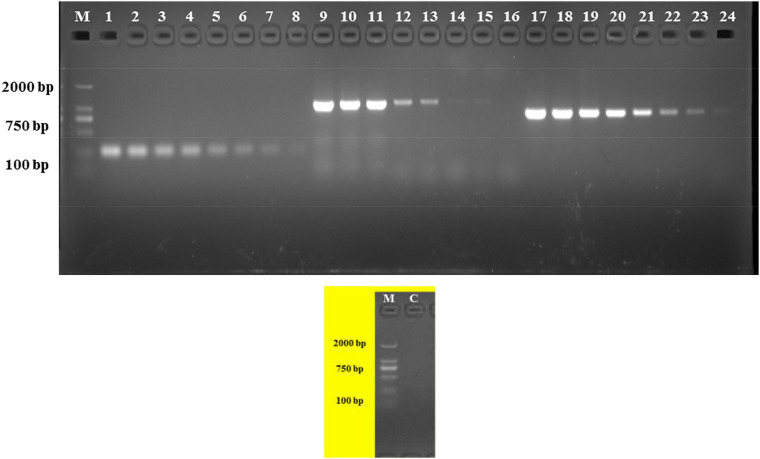
PCR detection sensitivity using dilutions of a pure culture of S. Albany strain SA48(32). Lane M: DSTM 2000 marker; Lane C: negative control (double-distilled H_2_O); lane 1–8, 9–16, and 17–24 primer sets based on group_27297, group_27289 and group_27286 genes, respectively (concentrations ranging from 2.8 × 10^7^ to 10^0^ CFU/mL).

The analytical sensitivity test showed that the detection limit of the primers designed based on the group_27297 and group_27286 primers was approximately 28 CFU/mL (Figure 4), whereas that of the group_29844 primer based on *Salmonella* serovar Hadar was approximately 2.3 × 10^4^ CFU/mL (Figure 2). Others showed a detection limit of 2.8–2.3 × 10^3^ CFU/mL (Figures 3, 4).

### Detection of Sereotype *Salmonella* in Artificially Contaminated Raw Pork Samples

Artificially contaminated raw pork samples were used to evaluate the sensitivity, specificity, and reliability of the primer sets designed based on serovar-specific genes. As shown in Table 2, the novel target-specific Albany serovar achieved a limit of detection as low as 1.04 × 10^3^–10^4^ CFU/g via PCR and target-specific Hadar serovar achieved a limit of detection as low as 1.16 × 10^4^–10^5^ CFU/g via PCR.

## Discussion

At present, the Kauffmann-White scheme of serotyping is still the most commonly used method for the identification of *Salmonella*. This conventional method is challenging in its complicated control of serum quality and lengthy, while the role of NAATs such as PCR is clear in their rapid identification of foodborne diseases. The usefulness of the DNA amplification method is predicated on the selection of the appropriate target sequence and specificity of the primer sets. A few serovar-specific fragments for *Salmonella* are available except for those related to genes of the O and H antigens such as *STM4495* for S. Typhimurium (Liu et al., 2012), *sdfI* for S. Enteritidis (De Freitas et al., 2010), *ISR2* for S. Infantis (Akiba et al., 2011), and *Newp2* for *S. Newport* (Bugarel et al., 2017), some of which may also be present in non-target strains due to the low specificity of targets—easily causing false-positive and false-negative results. Thus, additional novel specific molecular targets for *Salmonella* serovars must be identified and applied.

The comparative genomic method provides an available and efficient approach to obtain specific molecular targets for different pathogens. Unlike the complicated process of suppression subtractive hybridisation (requires restriction endonuclease, ligase, vector plasmid, and hybridisation) used for identifying specific targets to detect *Salmonella* serovar of Enteritidis and Pullorum (Agron et al., 2001; Li et al., 2009), the comparative genomic method only requires the collection of genomic sequences, classification target and non-target strains, and identification specific targets only present in target strains but absent in non-target strains by computers. Pan-genome analysis has been used to identify specific markers in bacteria, such as serotype, virulence, and antibiotic resistance, because bacterial phenotypes are usually associated with specific genes acquired through horizontal gene transfer (Buchanan et al., 2017; Laing et al., 2017). These genes are most frequently accessory genes in a bacterial species and are only present in bacterial strains that show corresponding phenotypes (McInerney et al., 2017). In order to obtain targets with high reliability and feasibility, we herein employed the pan-genome analysis approach to identify serovar-specific molecular targets for the detection of serogroup C2.

In a previous study, the *SNSL254_A2005* gene (hypothetical protein) was evaluated as a PCR target for *Salmonella* serogroup C2 detection and the results demonstrated that among 4 *Salmonella* C2 isolates of 2 serovars and 103 non-target isolates, the *SNSL254_A2005*-based PCR detection showed 100% inclusivity and 100% exclusivity (Liu et al., 2011). In our study, the percentage of genomic sequences of strains for the *SNSL254_A2005* gene presence in serogroup C2 and in other serogroups was 96.52% (555/575) and 2% (1/150), respectively, based on the gene presence/absence profile by pan-genome analysis. Thus, according to our results and previous studies, it can be concluded that the *SNSL254_A2005* gene is an effective specificity target for *Salmonella* serogroup C2 detection.

Currently, O and H antigen genes are ordinary targets for the detection of *Salmonella* serovars based on PCR (Yang et al., 2012; Maurischat et al., 2015). The major drawbacks of this approach are the utilisation of several targets for the detection of one serotype. In this study, we obtained seven novel single targets to detect Salmonella serovar Hadar and Albany. The HSR3 and Hadspe (hypothetical protein) genes were evaluated as PCR targets for *Salmonella* serovar Hadar detection and the results showed high specificity (100%) of this gene (Bugarel et al., 2017; Chiang et al., 2018). Compared to previous reports, the percentage of genomic sequences of strains for the four novel genes (group_20134, group_22774, group_29844, and group_29846) and HSR3 specific for serovar Hadar presence in target strains was higher (97.56%, 40/41) than in the Hadspe (82.93, 34/41), whereas in non-target serovars, the HSR3 genes were observed in serovars Paratyphi B and Tees (0.29%, 2/684). To the best of our knowledge, no primer set has been reported for the specific detection of serovar Albany. In the current study, the group_27286, group_27289, and group_27297 genes were identified to be specific to serovar Albany. The percentage of genomic sequences of strains for the gene presence in serovar Albany was 100% (15/15) and in other serovars was 0.14 (1/710), 0.14 (1/710), and 0.28 (2/710). These three genes were present in one strain for serovar Newport (0.4%, 1/248) and one strain for serovar Kentucky (0.85, 1/117) was also observed in the group_27297 gene. In addition, Newp and Newspe genes were evaluated as PCR targets for *Salmonella* serovar Newport detection in a previous report and the results showed almost all of tested strains belonged to serovar Newport, Hadar, Bovismorbificans, Kottbus, Blockley, Manhattan, Litchfield, Glostrup, and several strains belonged to serovar Virchow and Muenchen were amplified by Newp gene; in order to distinguish serovar Newport and Hadar, the Newspe marker was designed, which showed 93.3% (28/30) strains for Newport and only S. Blockey were amplified Newspe gene (Chiang et al., 2018). This result was also observed in our study. The percentage of genomic sequences of strains for the Newp gene in target and non-target strains was 87.5% (217/248) and 23.69% (113/477), respectively, and 87.8% (36/41) strains for S. Hadar also had this gene, but when the Newspe gene was added, although *S. Hadar* did not show a cross-reaction, there were still some serovars harbouring of the Newspe gene, in which the percentage of genomic sequences of strains for the Newp gene in target and non-target strains was 78.3% (188/248) and 23.69% (169/477), respectively. Based on our research, there is no ideal specific gene for S. Newport. The results suggest that some molecular targets previously considered specific may inevitably result in elimination with the expansion of genome databases. We also analysed the potential functions of serovar-specific genes. Consistent with previous reports (Kim et al., 2006; Liu et al., 2012; Zhang et al., 2018), all of the selected specific genes for the serovar are encoded hypothetical proteins or putative proteins. Therefore, future studies on the functions of the selected seven genes are necessary for a better understanding of *Salmonella* serovar Hadar and Albany.

The primers for the PCR assays were designed according to the sequences of the seven specific genes and specificity was verified using 158 isolates with 42 *Salmonella* serovars in China and other genera. All primer sets showed perfect specificity in the PCR assays (Table 1). We determined the detection limit of the PCR assays using seven primer sets and different concentrations of pure *Salmonella* cultures. The detection limit for primer sets to serovar Albany and Hadar were 2.8 × 10^1–3^ CFU/mL and 2.3 × 10^3–4^ CFU/mL, respectively (Figure 4); these results are in agreement with those reported by Kong et al. (2013), Chen et al. (2015), and Chin et al. (2017). The sensitivity, specificity, and reliability of the PCR were further confirmed in S. Albany and Hadar in artificially contaminated raw pork samples. The detection limit was 1.04 × 10^3–4^ CFU/g and 1.16 × 10^4–5^ CFU/g in raw pork samples, which was in the same range as that of other targets by PCR (Beaubrun et al., 2012). Therefore, it can be applied for rapid convenient, specific and sensitive detection of *Salmonella* serovar Albany and Hadar by PCR using seven serovar-specific genes in food.

## Conclusion

In conclusion, pan-genome analysis is an effective method to identify molecular targets specific to *Salmonella* serovars and the identified genes can be used as targets for molecular typing and identification of *Salmonella* serovars. The accuracy of the PCR results showed that it was a suitable method to explore the differences among serogroups. Future studies are needed to identify other single specific targets for the identification of other *Salmonella* serovars through pan-genome analysis for use as a new target for improved high-throughput detection methods crucial for the effective treatment and prevention of transmission from animal food to humans.

## Data Availability Statement

All datasets generated for this study are included in the article/Supplementary Material, further inquiries can be directed to the corresponding author/s.

## Author Contributions

QY contributed to design the experiments, analysed the data, and wrote the manuscript. QW, YD, and MC contributed to revise the manuscript. YS, FL, XX, CW, and BZ acquired the data. RP, SZ, JZ, XY, and LX played an important role in interpreting the results. All authors contributed to the article and approved the submitted version.

## Conflict of Interest

The authors declare that the research was conducted in the absence of any commercial or financial relationships that could be construed as a potential conflict of interest.

## Supplementary Material

Supplementary Figure 1 Distribution of pan-genome.

Supplementary Table 1 725 genomic sequences of Salmonella serovar were divided into serogroup C2 and others databanks.

Supplementary Table 2 Serovar-specific genes for Salmonella serogroups C2.
