# Supplementary figures and images for: Identification of Novel Sensitive and Reliable Serovar-Specific Targets for PCR Detection of Salmonella Serovars Hadar and Albany by Pan-Genome Analysis
Source: Front Microbiol. 2021 Mar 16;12:605984. doi: 10.3389/fmicb.2021.605984 (PMC8011537; doi:10.3389/fmicb.2021.605984)

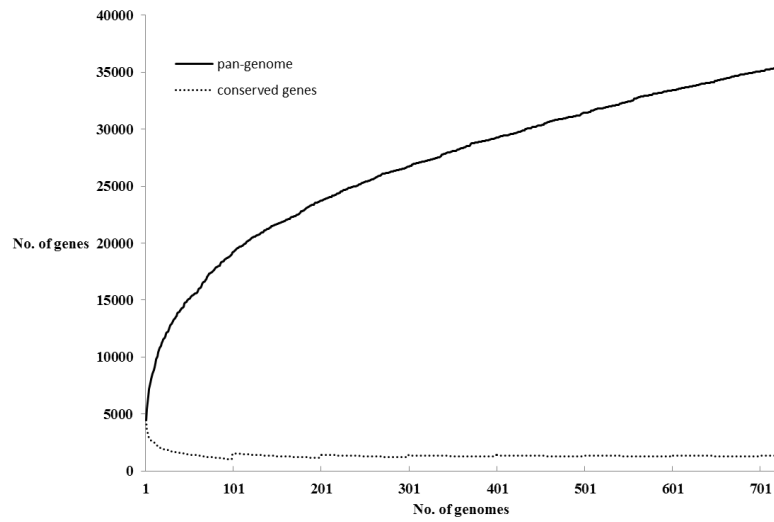

Figure S1 Distribution of pangenome

Supplement: Supplementary Figure 1 — Distribution of pan-genome. [file Image_1.pdf]
