# Supplementary material for: Identification of Novel Sensitive and Reliable Serovar-Specific Targets for PCR Detection of Salmonella Serovars Hadar and Albany by Pan-Genome Analysis
Source: Front Microbiol. 2021 Mar 16;12:605984. doi: 10.3389/fmicb.2021.605984 (PMC8011537; doi:10.3389/fmicb.2021.605984)
Supplement: Supplementary Table 2 — Serovar-specific genes for Salmonella serogroups C2. [file Table_2.docx]

Table S2 Serovar-specific genes for Salmonella serogroups C2

| serovar | Related gene | Annotation | | | Number of transmembrane | Signal peptide | Dscription |
| --- | --- | --- | --- | --- | --- | --- | --- |
|  |  | NR_annot | COG_annot* | eggNOG_annot |  |  |  |
| *S.Hadar* | group_20134 | hypothetical protein | S | / | / | / | / |
|  | group_22774 | putative protein | S | ATPase domain protein | / | / | / |
|  | group_29844 | hypothetical protein | / | / | / | / | / |
|  | group_29846 | hypothetical protein | / | / | 4 | / | Cell membrane protein |
| *S. Albany* | group_27297 | hypothetical protein | / | / | 1 | / | Cell membrane protein |
|  | group_27289 | hypothetical protein | V | type I restriction-modification system | / | / | / |
|  | group_27286 | hypothetical protein | S | / | 0 | Y | Secreted extracellular proteins |

* S: Function unknown, V: Defense mechanisms
